# The SUN2-nesprin-2 LINC complex and KIF20A function in the Golgi dispersal

**DOI:** 10.1038/s41598-021-84750-4

**Published:** 2021-03-08

**Authors:** Miki Hieda, Taizo Matsumoto, Mari Isobe, Sadamu Kurono, Kaneko Yuka, Satoshi Kametaka, Jing-Ya Wang, Ya-Hui Chi, Kenji Kameda, Hiroshi Kimura, Nariaki Matsuura, Shuji Matsuura

**Affiliations:** 1 Graduate School of Health Sciences, Ehime Prefectural University of Health Sciences, Ehime, 791-2101 Japan; 2 Graduate School of Medicine and Health Science, Osaka University, Osaka, 565-0871 Japan; 3 Nagoya University Graduate School of Medicine, Nagoya, 461-0047 Japan; 4 Laboratory Chemicals Division, Wako Pure Chemical Industries, Ltd, Osaka, 540-8605 Japan; 5 Institute of Biotechnology and Pharmaceutical Research, National Health Research Institutes, Zhunan Township, Taiwan; 6 ADRES, Ehime University, Ehime, 791-0204 Japan; 7 Institute of Innovative Research, Tokyo Institute of Technology, Yokohama, 226-8501 Japan; 8 Osaka International Cancer Institute, Osaka, 541-8567 Japan; 9 Present Address: Graduate School of Medicine and Dental Sciences, Kagoshima University, Kagoshima, 890-8544 Japan

**Keywords:** Cell biology, Cell polarity, Organelles

## Abstract

The morphology of the Golgi complex is influenced by the cellular context, which strictly correlates with nuclear functions; however, the mechanism underlying this association remains elusive. The inner nuclear membrane SUN proteins, SUN1 and SUN2, have diverse functions together with the outer nuclear membrane nesprin proteins, which comprise the LINC complex. We found that depletion of SUN1 leads to Golgi complex dispersion with maintenance of ministacks and retained function for vesicle transport through the Golgi complex. In addition, SUN2 associates with microtubule plus-end-directed motor KIF20A, possibly via nesprin-2. KIF20A plays a role in the Golgi dispersion in conjunction with the SUN2-nesprin-2 LINC complex in SUN1-depleted cells, suggesting that SUN1 suppresses the function of the SUN2-nesprin-2 LINC complex under a steady-state condition. Further, SUN1-knockout mice, which show impaired cerebellar development and cerebellar ataxia, presented altered Golgi morphology in Purkinje cells. These findings revealed a regulation of the Golgi organization by the LINC complex.

## Introduction

The nucleus is surrounded by a nuclear envelope (NE) comprising an inner and an outer nuclear membranes (INM and ONM, respectively). The LINC (linker of nucleoskeleton and cytoskeleton) complex consists of the INM-spanning SUN domain-containing proteins, designated SUNs, and the ONM-spanning KASH domain-containing proteins, designated nesprins. In mammalian genome, five genes that encode SUN proteins (SUN1–5) are present. SUN1 and SUN2 are widely expressed in mammalian somatic cells^1,2^, whereas the expression of SUN3, SUN4 (also known as sperm associated antigen 4, SPAG4), and SUN5 (also known as SPAG4-like, SPAGL) is largely, but not entirely, restricted to the germ cells^3–6^. There are six KASH-containing proteins in mammals, including four nesprins^7^, KASH5, and the Lymphoid Restricted Membrane Protein (LRMP, also called Jaw1)^8,9^. KASH5 and LRMP/Jaw1 are expressed in germ cells and lymphoid cells, respectively. SUNs and nesprins physically interact with each other in the lumen of the NE^1^. SUN proteins interact with lamins and chromatin, whereas nesprins associate with cytoskeletal components including microtubule (MT) motors and actin filaments^7^. Thus, the LINC complex directly connects the cytoskeleton and nucleoskeleton to form a scaffold for diverse functions including nuclear migration, shaping, and positioning, maintenance of the centrosome-nucleus connection, mechanotransduction, DNA repair, cell migration, and moving chromosomes within the nucleus during meiosis^10^.

In most vertebrate cells, individual Golgi stacks are laterally connected in a juxtanuclear array, termed the Golgi ribbon, in close association with the MT-organizing center^11^. The structure and positioning of the Golgi complex are highly dynamic and dramatically changes during several cellular processes such as cell cycle progression, migration, and differentiation. These processes strictly correlate with nuclear functions; however, the mechanism underlying the association between the Golgi morphology and nuclear functions remains elusive.

KIF20A, originally identified as Rabkinesin-6 and also referred to RabKIFL or MKLP2, interacts with Rab6 and belongs to the family of plus-end-directed MT motor proteins (i.e., kinesins)^12^. Rab6 is the most abundant Golgi-associated Rab protein^13^ and functions in the biogenesis of Golgi vesicles^14^. Endogenous KIF20A is localized to the Golgi complex in interphase cells and plays a role in Golgi complex dynamics^12,15^. Juxtanuclear positioning of the Golgi complex is the balanced outcome of minus- and plus-end-directed MT-dependent motors. In normal cells, the minus-end-directed MT motor dynein dominates plus-end-directed motor activities under a steady-state condition. On the other hand, the Golgi is dispersed into small structures scattered throughout the cytoplasm, in cells expressing high amounts of KIF20A^12^. The present study demonstrates that the LINC complex plays a role in maintaining Golgi integrity together with KIF20A. This mechanism may connect Golgi dynamics with nuclear features because SUN proteins interact with chromatin and the nuclear lamina.

## Results

### SUN1 is required for perinuclear organization of the Golgi complex

**Figure 1:**
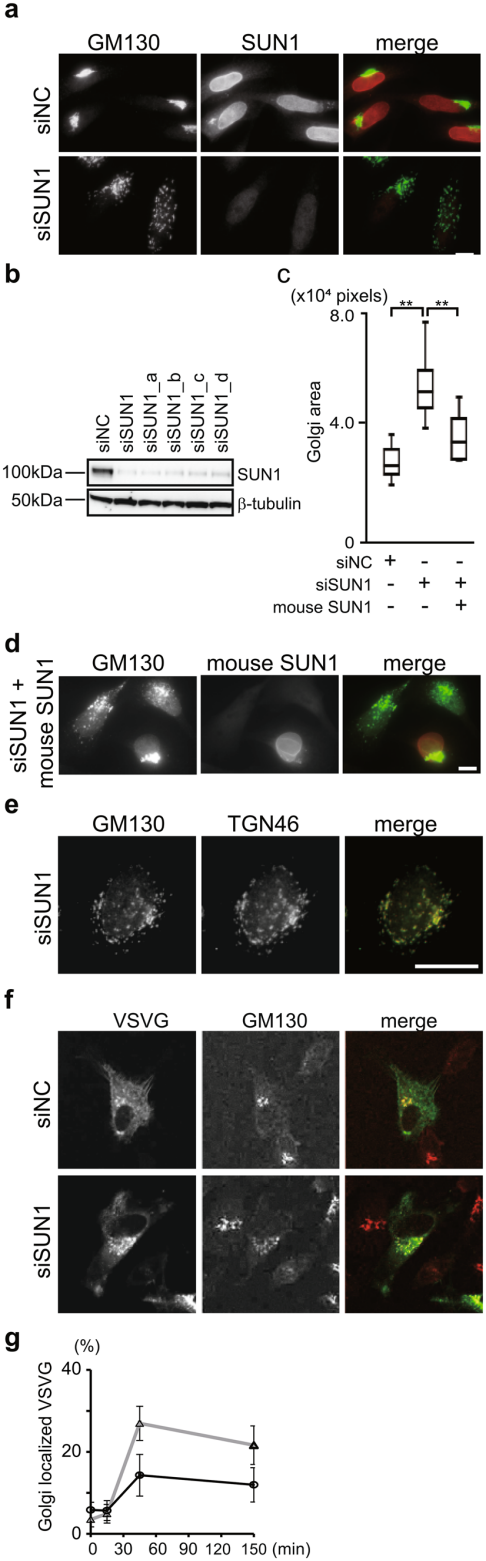
SUN1 is associated with Golgi complex morphology. (**a**) HeLa cells were transfected with siSUN1 or siNC. After fixation, cells were stained with anti-GM130 mAb (green) and anti-SUN1 pAb (red). Note that, siSUN1 contains four siRNAs, siSUN1_a, siSUN1_b. siSUN1_c, and siSUN1_d. Bar, 10 µm. (**b**) Cells were transfected with indicated siRNA. SUN1 and β-tubulin protein expressions were analyzed. (**c**) Box-and-whiskers plots represent GM130 labeled Golgi complex area per cell**.** ** P < 0.01, compared with control cells. (**d**) Cells were transfected with siSUN1. After 24 h, cells were transfected with Myc-tagged mouse SUN1 and incubated for 24 h. The cells were then stained with anti-GM130 pAb and anti-Myc mAb. Bar, 10 µm. (**e**) HeLa cells were transfected with siSUN1 and stained with anti-GM130 mAb and anti-TGN46 pAb. Bar, 10 µm. (**f**) GFP-tsVSVG was transfected into control or SUN1-knockdown cells. After shifting to the permissive temperature, cells were incubated for 2.5 h. VSVG (green) and the Golgi complex (red) were detected using GFP and anti-GM130 mAb, respectively. (**g**) The ratio of VSVG intensity in the GM130 positive Golgi area to total VSVG in the cell is shown. The gray line with triangle and the black like with the circle represents control and SUN1-depleted cells, respectively.

We previously showed that depletion of SUN1, but not SUN2, severely suppresses directional cell migration^16^. Because the Golgi complex confers overall cell polarity to the cell, we examined the effects of SUN1 depletion on Golgi morphology using HeLa cells, which exhibit the perinuclear-accumulated Golgi complex. Transfection with a pool of four small interfering RNAs (siRNAs) against SUN1 (siSUN1) decreased SUN1 protein expression (Fig. 1a,b)^16^. In addition, transfection of siSUN1, but not negative control siRNA (siNC), drastically dispersed the Golgi complex throughout the cytoplasm (Fig. 1a and Fig. S1 for low magnification field of view) as detected with an antibody specific for the cis-Golgi marker GM130 (Golgi matrix protein 130). Upon depletion of SUN1, the GM130 positive area increased approximately twofold (Fig. 1c). Transfection with each single siRNA against SUN1 (siSUN1_a, siSUN1_b, siSUN1_c, and siSUN1_d) also decreased the protein expression (Fig. 1b, and Fig. S2a) and induced the Golgi dispersion (Fig. S2b,c). The dispersed Golgi structure was rescued by the expression of siRNA-resistant mouse SUN1 (Fig. 1c,d). These results confirmed that Golgi dispersal is not a non-specific effect of siRNA transfection. Dispersal of the Golgi complex triggered by SUN1 knockdown was not specific to HeLa cells but was also observed in SUN1-depleted non-cancerous MCF10A (Fig. S2d,e). Moreover, the dispersed GM130 molecules in the SUN1-depleted cells were situated in close proximity to the trans-Golgi marker TGN46 (Fig. 1e), suggesting that these Golgi complexes in the SUN1-knockdown cells maintain ministacks, which remain competent for posttranslational processing and protein secretion^17^. To investigate vesicle transport through the Golgi complex, we used a temperature-sensitive mutant of vesicular stomatitis virus glycoprotein (tsVSVG). This secretory cargo accumulates in the ER at non-permissive temperatures due to a reversible folding defect (time 0). At the permissive temperature, correctly folded tsVSVG is transported from the ER through the Golgi complex (Fig. 1f). The quantitative data indicated the vesicle transport through the Golgi complex in SUN1 depleted cells, though these cells decreased the amount of tsVSVG arriving in the Golgi complex (Fig. 1g).

As another example of Golgi fragmentation, depletion of GM130 causes fragmentation and defective functioning of the Golgi complex^18^. However, the immunofluorescent data showed that SUN1 knockdown did not alter the amount of GM130. Furthermore, it is known that the centrosome remains at the perinuclear region of SUN1-depleted cells^19^. Therefore, perturbation of centrosome organization is not responsible for the Golgi dispersal induced by SUN1 depletion.

### SUN2 functions in Golgi dispersion in SUN1-depleted cells

**Figure 2:**
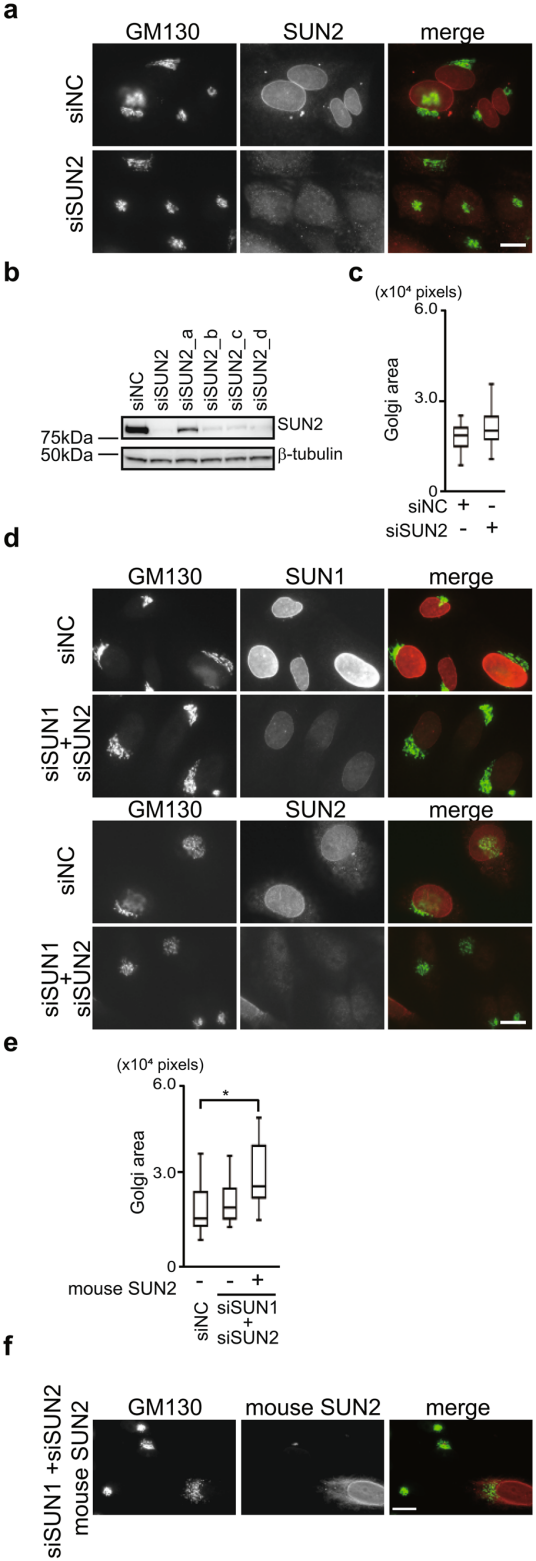
SUN2 functions in dispersal of the Golgi complex. (**a**) Cells were transfected with a pool of siSUN2 or siNC and stained with anti-GM130 mAb (green) and anti-SUN2 pAb (red). Bar, 10 µm. Note that, siSUN2 contains four siRNAs, siSUN2_a, siSUN2_b. siSUN2_c, and siSUN2_d. (**b**) Cells were transfected with indicated siRNA. SUN2 and β-tubulin protein expressions were analyzed. (**c**) Box-and-whiskers plots represent GM130 labeled Golgi complex area in SUN2 depleted cells**.** (**d**) Cells were transfected with siSUN1 and siSUN2 or siNC. The cells were then stained with anti-SUN1 or anti-SUN2 pAb (red) and anti-GM130 mAb (green). Bar, 10 µm. (**e**) Box-and-whiskers plots represent GM130 labeled Golgi complex area in indicated cells**.** * P < 0.05, compared with siNC transfected cells. (**f**) Cells were transfected with siSUN1 and siSUN2. After 12 h, cells were transfected with Myc-tagged mouse SUN2 and incubated for 36 h. The cells were then stained with anti-Myc mAb and anti-GM130 pAb. Bar, 10 µm.

Mammalian somatic cells mainly express two SUN proteins, SUN1 and SUN2. We next investigated the involvement of SUN2 in Golgi organization because SUN1 and SUN2 have both redundant and distinct functions^20–25^. Cells were transfected with a pool of siRNA against SUN2 (siSUN2), and knockdown was confirmed by immunofluorescence staining and Western blotting (Fig. 2a,b)^23^. Knockdown efficiency of each siRNA (siSUN2_a, siSUN2_b, siSUN2_c, and siSUN2_d) was also confirmed in Western blotting (Fig. 2b and Fig. S2f). In contrast to the effects of SUN1 depletion (Fig. 1), SUN2 knockdown did not trigger Golgi complex dispersal (Fig. 2a,c). This finding indicates that SUN1 and SUN2 play distinct roles in Golgi maintenance. To assess the effects of SUN2 depletion on Golgi dispersion triggered by SUN1 knockdown, SUN1 and SUN2 were simultaneously depleted. SUN1/SUN2 double-knockdown cells showed the perinuclear Golgi complex with a normal morphology (Fig. 2d,e), indicating that SUN2 is required for Golgi dispersal induced by SUN1 depletion and SUN1 is dispensable for the perinuclear Golgi structure under a steady state. To confirm the specificity of siSUN2, siRNA-resistant mouse SUN2 was transfected into the SUN1/SUN2 double-knockdown cells. Mouse SUN2 expression induced Golgi dispersal in these cells (Fig. 2e,f) showing the specificity of siSUN2 and a function of SUN2 in Golgi dispersal induced by SUN1 depletion.

Next, to investigate the underlying mechanism of the Golgi dispersion in SUN1-depleted cells, we evaluated the cell cycle in SUN1 and/or SUN2 knockdown cells because the cell cycle has a major role in Golgi integrity. However, the depletion of SUN proteins did not affect the cell cycle distribution (Fig. S3).

### Nesprin-2 is essential for Golgi dispersal induced by SUN1 depletion

**Figure 3:**
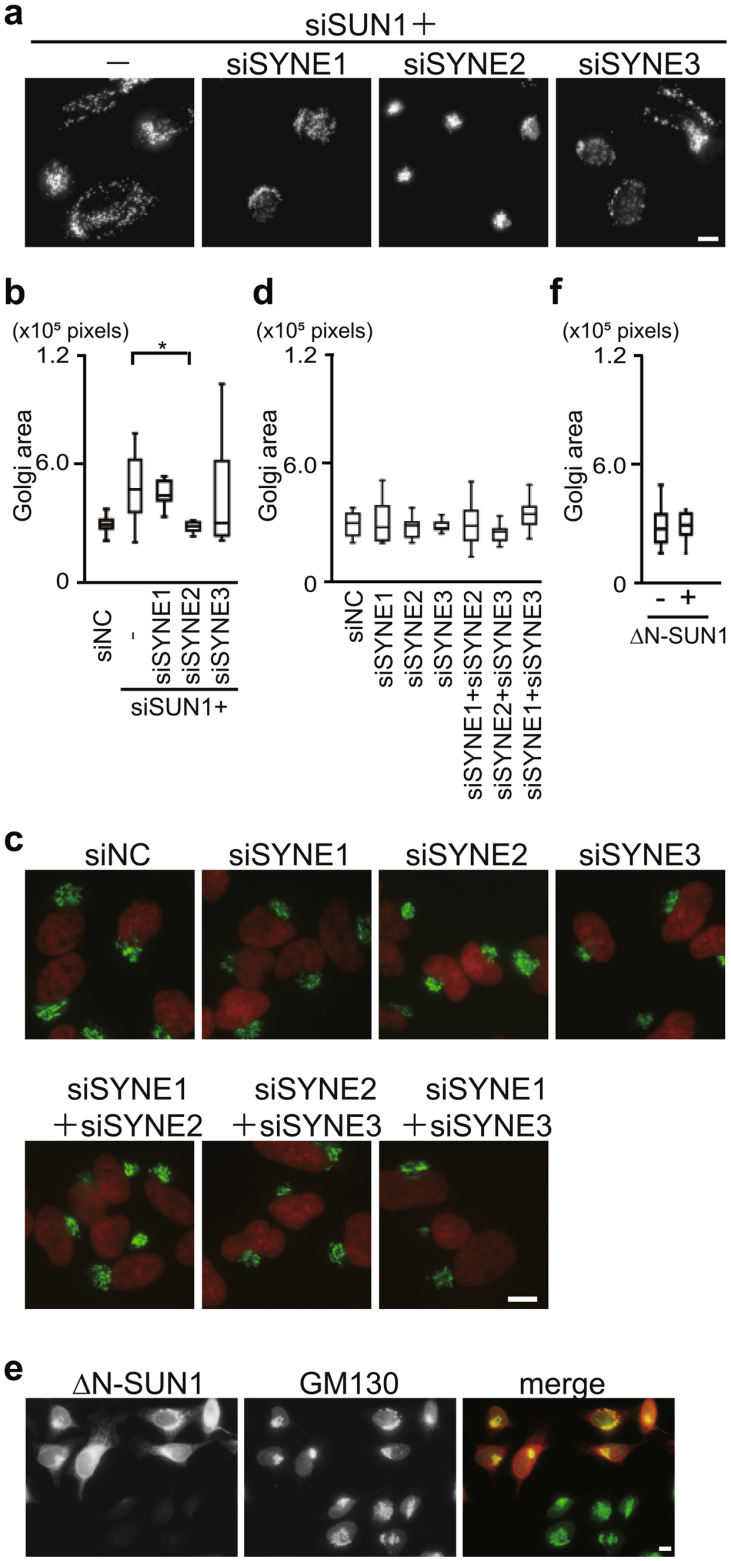
Nesprin-2 functions in dispersal of the Golgi complex but not in the steady state perinuclear Golgi structure. (**a**) Cells were transfected with siSUN1 and indicated siRNAs including siSYNE1, siSYNE2, or siSYNE3, which targets nesprin-1, nesprin-2, and nesprin-3, respectively. The cells were then stained with anti-GM130 mAb. Bar, 10 µm. (**b**, **d**) Box-and-whiskers plots represent GM130 labeled Golgi complex area. * P < 0.05. (**c**) Cells were transfected with indicated siRNA and stained with anti-GM130 mAb (green) and DAPI (red). (**e**) Cells were transfected with HA-tagged ΔN-SUN1 and stained with anti-HA mAb (red) and anti-Golgi pAb (green). (**f**) Box-and-whiskers plots represent GM130 labeled Golgi complex area in HA-tagged ΔN-SUN1 transfected or non-transfected cells.

It is well established that INM-localized SUN proteins, together with ONM-localized nesprins, function as the LINC complex. To examine which nesprin molecules(s) play a role in the SUN2-dependent Golgi dispersal induced by SUN1 depletion, nesprin-1, -2, or -3 were knocked down in conjunction with SUN1 depletion; nesprin-4 expression is not detectable in HeLa cells^26^. Knockdown of nesprin was confirmed by RT-PCR (Fig. S4a). Remarkably, nesprin-2 knockdown, but not nesprin-1 or -3 knockdown, suppressed Golgi dispersal in the SUN1-depleted cells (Fig. 3a,b), indicating that nesprin-2 but not the other nesprins is essential for Golgi dispersal induced by SUN1 depletion. These results demonstrate that the SUN2-nesprin-2 LINC complex functions in Golgi dispersal in SUN1-depleted cells.

Next, to examine whether nesprins are dispensable for the juxtanuclear Golgi organization in a steady-state, each nesprin was depleted using the specific siRNA. As expected from the SUN1/SUN2 double-knockdown cells, the depletion of any nesprin did not cause apparent alterations to the Golgi morphology (Fig. 3c,d). However, these could have been the result of nesprin-1, -2, and -3 playing overlapping roles in perinuclear Golgi organization. Thus, combinatorial depletion using two　siRNAs against nesprins was performed and the result showed that depleting any combination still failed to affect Golgi morphology (Fig. 3c,d), indicating that all of the nesprins, i.e., nesprin-1, -2, and -3, are not essential for juxtanuclear Golgi organization under a steady-state condition.

Further, to examine whether the interactions of SUNs and nesprins are dispensable for the perinuclear Golgi organization, we used a dominant-negative SUN domain construct (ΔN-SUN1), which contains transmembrane and SUN domains (i.e., a.a. 205–785) and disrupt the interactions between endogenous SUNs and nesprins^27^. Overexpression of ΔN-SUN1 did not affect the perinuclear Golgi morphology (Fig. 3e,f). Therefore, we conclude that the interactions of SUNs and nesprins, i.e., LINC complexes, are not required for the perinuclear Golgi organization under a steady-state condition.

### KIF20A is required for Golgi dispersal induced by SUN1 depletion

**Figure 4:**
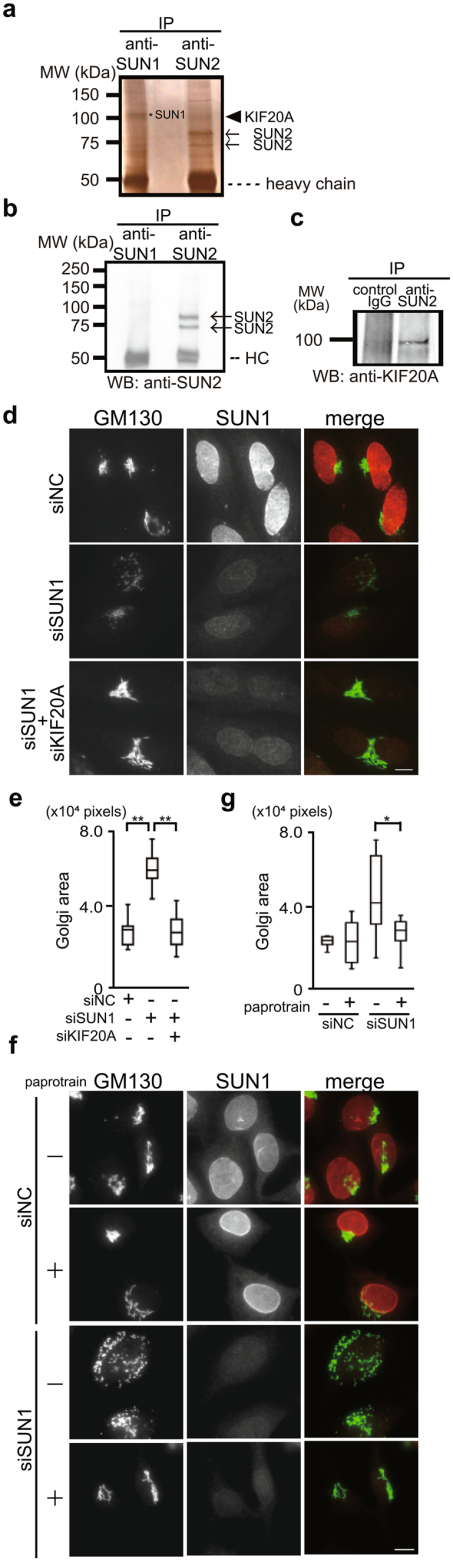
KIF20A is essential for Golgi scattering induced by SUN1 depletion. (**a**) SUN1- and SUN2-containing protein complexes were precipitated using anti-SUN1 or anti-SUN2 pAb, resolved by SDS-PAGE, and subjected to silver staining; the bands indicated by arrows, an arrowhead, and an asterisk were analyzed by LC–MS/MS. The bands indicated by arrows with molecular weights of approximately 75 kDa are two SUN2 splicing variants, RefSeq NM_001199580 and RefSeq NM_001199579. (**b**) Immunoprecipitation was performed using anti-SUN1 (lane 1) or anti-SUN2 pAb (lane 2) and analyzed by Western blotting using anti-SUN2 pAb. (**c**) Immunoprecipitation was performed using normal rabbit IgG (lane 1) or anti-SUN2 pAb (lane 2) and analyzed by Western blotting using anti-KIF20A pAb. (**d**) and (**e**) Cells were transfected with siSUN1 and siRNA against KIF20A (siKIF20A). The cells were then stained with anti-SUN1 pAb and anti-GM130 mAb. Bar, 10 µm**.** (**e**) Box-and-whiskers plots represent GM130 labeled Golgi complex area**.** ** P < 0.01. (**f**) and (**g**) Cells were transfected with siSUN1 and incubated for 36 h. Paprotrain was then added. After 12 h, the cells were fixed and stained with anti-SUN1 pAb and anti-GM130 mAb. Bar, 10 µm. (**g**) Box-and-whiskers plots represent GM130 labeled Golgi complex area**.** * P < 0.05.

SUN2 and nesprin-2 play a role in the dispersal of the Golgi complex in SUN1-depleted cells (Fig. 2). To elucidate the underlying mechanisms responsible for this phenomenon, we attempted to identify proteins that specifically interact with the SUN2-nesprin-2 LINC complex by co-immunoprecipitation. An anti-SUN2 antibody precipitated several bands including two SUN2 splicing variants (Fig. 4a, arrows, and Fig. S4b) and several nesprin-2 variants, which were verified by liquid chromatography with mass spectrometric analysis (LC–MS/MS) and Western blotting (Fig. 4b, and Fig. S4c). In addition, the anti-SUN2 antibody also precipitated a 100-kDa band (Fig. 4a, right, arrowhead). This protein was identified as KIF20A by LC–MS/MS analysis and Western blotting (Fig. 4c). KIF20A was not detected in the immunoprecipitates obtained with control IgG (Fig. 4c, and Fig. S4d). Note that a 100-kDa band in anti-SUN1 precipitates (Fig. 4a, left, asterisk) was SUN1, which was confirmed by LC–MS/MS and Western blotting.

KIF20A has been reported to function in the Golgi organization. Therefore, to examine the function of KIF20A in Golgi complex dispersion caused by SUN1 depletion, KIF20A were depleted together with SUN1 using siRNA. This treatment led to a > 90% reduction in the level of KIF20A mRNA (Fig. S4a) and markedly suppressed Golgi dispersal in the SUN1-depleted cells (Fig. 4d,e). Note that because KIF20A functions in both Golgi maintenance and mitotic exit, its depletion inhibits cytokinesis and results in the accumulation of multinucleated cells^28,29^. Moreover, a KIF20A inhibitor, paprotrain, also suppressed Golgi dispersal in the SUN1-depleted cells (Fig. 4f,g). These results indicate that KIF20A is essential for the dispersion observed in the SUN1-depleted cells.

### SUN1 is associated with Golgi organization in vivo

**Figure 5:**
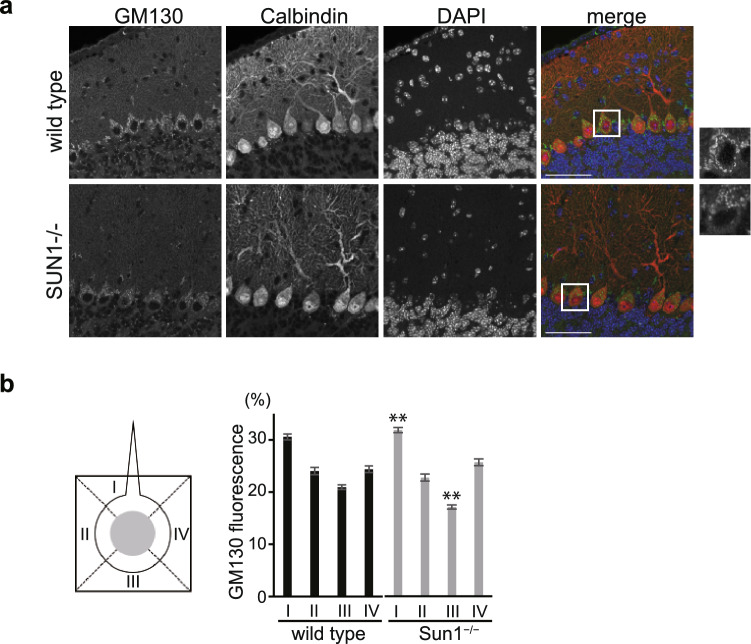
SUN1 functionally associates with Golgi organization in vivo. (**a**) The cerebella from 30-day old male wild-type and Sun1-knockout mice were stained using anti-GM130 pAb and anti-calbindin (a Purkinje cell marker protein) mAb. Bar, 20 µm. The panels on the right are enlarged images of the indicated region in the adjacent panels on the left. (**b**) Golgi distribution was quantified using GM130 staining as the average percentage of total cellular fluorescence in each of the four quadrants (shown at left). The results were obtained from the quantification of 10 < Purkinje cells in each genotype. Data are presented as the mean ± sem, ** P < 0.01, compared with the same region in wild-type tissues.

We next looked into the Golgi complex in vivo. *Sun1*^*−/−*^ mice are born normally but exhibit infertility and impaired hearing^30–32^. Sun1 is selectively expressed in the Purkinje cells of the cerebellum in wild-type mice, and Sun1-knockout mice later present with impaired cerebellar development and cerebellar ataxia^33^. Thus, the Golgi structure in the Purkinje cells from 30-day-old Sun1-knockout mice was investigated. The Purkinje cells had an elaborate Golgi ribbon, with Golgi elements extending around the nucleus of the cell soma (Fig. 5a upper, green). The distribution topology of the Golgi complex in Sun1^-/-^ cells was different from that in the wild-type cells (Fig. 5a, right panels). In addition, the staining intensity of the Golgi in the *Sun1*^*−/−*^ Purkinje cells was weaker than in the wild-type mice (Fig. 5a lower, green). Golgi distribution was then quantified as reported by Liu et al*.*^18^. In the Purkinje cells from the wild-type mice, the Golgi was slightly enriched at the apical pole (region I) relative to other regions (Fig. 5b). Interestingly, *Sun1*^*−/−*^ tissues showed a more uneven distribution of the Golgi complex than the wild types. GM130 signals in region III were decreased and localization of the Golgi complex in region I was increased instead (Fig. 5b). The loss of Sun1 altered the position of the Golgi complex in the soma of the Purkinje cells, demonstrating that Sun1 is important for maintaining Golgi organization in vivo.

## Discussion

**Figure 6:**
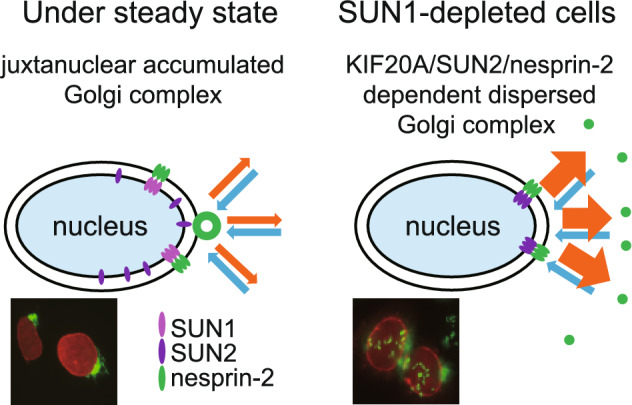
Working model for the regulation of the Golgi organization by LINC complex. Left, The minus-end-directed MT motor activity, i.e., dynein (blue), dominates plus-end-directed motor activity under a steady-state condition, and juxtanuclear accumulation of the Golgi complex is observed. Right, In SUN1-depleted cells, KIF20A is activated or suppression of KIF20A activity is cancelled, and KIF20A activity dominates dynein activity. An explanation for the inhibition of KIF20A activity in the presence of SUN1 is that LINC complex formations are biased towards SUN1 inclusion and SUN2/nesprin-2 LINC complex formation is suppressed. In response to SUN1 depletion, incorporation of nesprin-2 in the SUN1-LINC complex decreased and SUN2/nesprin-2 LINC complexes are formed. As a result, functions of KIF20A acting with a SUN2/nesprin-2 LINC complex becomes predominant (orange) over the minus-end-directed motor activities (blue), and the Golgi complex is dispersed.

This study showed that the LINC complex component SUN1 functions in the maintenance of the proper Golgi structure in vivo and in vitro. SUN2 and nesprin-2 play a role in Golgi dispersal in conjunction with KIF20A in SUN1-depleted cells, although SUN1 is dispensable for perinuclear Golgi integrity. These results suggest that SUN1 suppresses SUN2/nesprin2/KIF20A functions under a steady state, i.e., in both SUN1 and SUN2 expressing cells (Fig. 6).

Juxtanuclear positioning of the mammalian Golgi ribbon is the balanced outcome of minus- and plus-end-directed MT-dependent motor activities^34^. The minus-end-directed MT motor dynein dominates plus-end-directed motor activity under a steady-state condition, whereas most cells express several plus-end directed motors including KIF20A. This study showed that the function of KIF20A in cooperation with the SUN2/nesprin-2 LINC complex governs Golgi dispersion in SUN1-depleted cells. Presently, how SUN1 suppresses KIF20A functions under a steady state is unknown, but several mechanisms can be envisaged.

First, SUN1 may repress SUN2/nesprin2 LINC complex formation resulting in decreased KIF20A recruitment to the juxtanuclear area or suppressed loading of the Golgi complex to KIF20A. Mammalian somatic cells mainly express two SUN-domain proteins, SUN1 and SUN2, and several KASH domain-containing proteins such as nesprin-1, nesprin-2, and nesprin-3. Biochemical studies show that SUN and KASH domains interact promiscuously^35–39^. However, several reports have revealed that the interactions between them are biased: SUN1 is more efficiently incorporated into LINC complexes than SUN2 under normal growth conditions^40^, and SUN2 binding to KASH is weaker than SUN1^35^. These results imply that SUN1 suppresses the SUN2-nesprin2 LINC complex assembly sequestering KASH domain-containing proteins away from SUN2 (Fig. 6, left), and thus SUN1 depletion induces SUN2/nesprin-2 LINC complex formation, which plays a role in Golgi dispersal in conjunction with KIF20A (Fig. 6, right).

Alternatively, SUN1 may indirectly suppress KIF20A. SUN2 activates the small GTPase RhoA, and SUN1 antagonizes SUN2 LINC complexes and inhibits RhoA activation^24^. Because RhoA participates in actin and microtubule dynamics and microtubule stabilization by α-tubulin acetylation promotes the association with microtubule motors^41,42^, KIF20A activity might be indirectly regulated by SUN proteins via microtubule stabilization.

Findings from the present study have three important physiological implications. First, disruption of the Golgi architecture and function are widely observed in neurodegenerative diseases including Alzheimer’s and Parkinson’s disease, and reciprocally, several papers have reported that Golgi dysfunction can play a causative role in neurodegeneration^43^. Sun1-knockout mice manifest cerebellar ataxia^33^, while the underlying mechanism has been unknown. In this study, we show that Sun1 plays a role in maintaining the Golgi organization in vivo and in vitro. Therefore, the impaired Golgi complex induced by SUN1 depletion may trigger the above pathological sign. In addition, since it is known that Golgi fragmentation under physiological conditions, the LINC complex may function in regulating Golgi complex morphology under a broad range of conditions.

Second, mammalian somatic cells express two kinds of SUN protein, SUN1 and SUN2, and three or four nesprin proteins, and it has been believed that each LINC complex is formed via promiscuous interactions between the SUN and nesprin protein families. However, our results suggest a compositional regulation of the SUN–KASH hexamer formation, and this regulation may control the Golgi complex morphology.

Last, it is known that the LINC complex functions in mechanotransduction^44^ and meiotic telomere clustering^45,46^, in which it mediates cytoplasmic forces or signaling from the cytoplasm to the nucleus. In contrast, the present study revealed that LINC complexes regulate the morphology of a cytoplasmic organelle, suggesting that nuclear features such as histone modification or transcriptional activity may be relayed to the Golgi structure via LINC complexes and coordinate the functions of both the cytoplasm and the nucleus.

## Methods

### Antibodies and solutions

Rabbit GM130 (also known as GOLGA2, Golgin subfamily A member 2)-specific pAb (HPA021799) was purchased from Sigma Aldrich (St. Louis, MO, USA). Mouse anti-GM130 mAb (clone 35) was from Becton Dickinson and Company (Franklin Lakes, NJ, USA). Anti-Myc-tag mouse ascites fluid (clone 9E10, SAB4300605), mouse anti-Golgi 58 k mAb (G2404), mouse anti-calbindin (C9848), and rabbit anti-SUN1 pAb (HPA008346) were also from Sigma Aldrich. Sheep anti-TGN46 pAb (AHP500G) was from AbD Serotec (Oxford, UK). Anti-SUN2 pAb (#06–1038) was purchased from Millipore (Burlington, MA, USA). Rabbit anti-KIF20A pAb (ab70791) was obtained from Abcam (Cambridge, United Kingdom). Rat anti-HA mAb (3F10) was from Roche (Basel, Switzerland). Rabbit anti-GFP pAb (code No. 598) was from Medical and Biological Laboratories (Aichi, Japan). KIF20A inhibitor, paprotrain, was purchased from Sigma Aldrich and used at a final concentration of 20 µM^47^.

### Cell culture, transfection, immunofluorescence, and quantitative analysis of Golgi dispersion

HeLa cells were obtained from the JCRB cell bank (Japanese Collection of Research Bioresources, Japan) and grown in Dulbecco’s modified Eagle’s medium, low glucose (Fujifilm Wako Pure Chemical Corporation., Osaka, Japan) supplemented with 10% fetal calf serum at 37 °C in a 10% CO_2_ atmosphere. HeLa cells were used throughout this study unless otherwise stated. The human mammary epithelial cell line MCF10A (CRL-10317) was obtained from ATCC (Manassas, VA, USA) and cultured as previously described^48^. Transfection was performed using Lipofectamine 2000 (Invitrogen, Carlsbad, CA, USA) or Gene Juice (Merck Millipore, Darmstadt, Germany) in accordance with the manufacturer’s instructions. Immunofluorescence was performed as described previously^49^ using appropriate primary and secondary antibodies (Jackson ImmunoResearch Laboratories, Soham, UK), and coverslips were mounted using Prolong Gold Antifade Reagent with 4′, 6-diamidino-2-phenylindole (DAPI) (Life Technologies, Waltham, MA, USA) or stained with Hoechst 33,342 and mounted using Fluorescent Mounting Medium (S3023; Dako, Tokyo, Japan). Cells were viewed using an Olympus IX81 with Plan Apo 60 × /NA1.4 or Olympus BX53 with UPlanS Apo 40 × /NA 0.95 objective lens. To quantify the Golgi dispersion, GM130-staining areas were analyzed using ImageJ. The gray scale were binarized, then areas of GM130 staining per cells were measured (n > 10 cells). P values were calculated using the Student t test.

### Plasmids

Myc-tagged mouse SUN1 encoding 913 amino acids (NM_024451) and Myc-tagged mouse SUN2 were purchased from Origene (Rockville, MD, USA). GFP-tagged temperature-sensitive VSVG (tsVSVG) was a gift from Jennifer Lippincott-Schwartz^50^. The ΔN-SUN1 expression plasmid was constructed by cloning positions 205–785 of human SUN1 (NM_001130965.2) between the EcoRI and XhoI restriction sites of the pcDNA3.0 vector tagged with HA at the N-terminus^27^.

### Vesicle transport assay from the ER to the cell surface using tsVSVG

Cells were transfected with GFP-tsVSVG. This secretory cargo accumulates in the ER at non-permissive temperatures due to a reversible folding defect. When the temperature is shifted to the permissive temperature, correctly folded VSVG is rapidly transported from the ER through the Golgi complex to the cell surface. After shifting to the permissive temperature, cells were incubated for the indicated time, and VSVG and the Golgi complex were detected using GFP and anti-GM130 mAb, respectively. The transport activity was quantified as the ratio of VSVG intensity in the GM130 positive Golgi area to the total VSVG intensity in the cell using ImageJ Fiji software.

### siRNA-mediated knockdown

Predesigned siRNA pools (siGenome SMARTpool; each siRNA pool contains four siRNAs) against SUN1 (UNC84A), SUN2 (UNC84B), KIF20A, SYNE1 (nesprin-1), or SYNE2 (nesprin-2) were obtained from Thermo Fisher Scientific (Waltham, MA, USA)^16,23^. siRNA oligos against SYNE3 (nesprin-3) were designed by and obtained from Nippon Gene (Tokyo, Japan). The sequences of the siRNA pools targeting SUN1 and SUN2 were described previously^16^ and others are listed in Table S1. Negative control siRNA (siGENOME non-targeting siRNA pool, a mixture of four nontargeting siRNAs) was also obtained from Thermo Fisher Scientific. siRNAs against cells were transfected with targeting siRNA or a non-targeting siRNA pool (Thermo Fisher) using Lipofectamine RNAi MAX (Invitrogen). All siRNAs were used at a final concentration of 10 nM. Cells were fixed or harvested at 48 h (HeLa cells) or 72 h (MCF10A cells) after transfection. For rescue experiments, cells were transfected with indicated siRNA. After 24 h, cells were transfected with Myc-tagged mouse SUN1 or Myc-tagged mouse SUN2, and incubated for 24 h. To verify the knockdown efficiency of nesprin-1 to -3 and KIF20A, the expression of the corresponding mRNA was detected by RT-PCR using the appropriate primer sets (Table S2). Others were verified by Western blotting or immunofluorescence.

### Immunoprecipitation and Western blotting

For immunoprecipitation using anti-SUN1 and anti-SUN2 pAbs, HeLa cells were washed three times with ice-cold PBS (phosphate-buffered saline) and harvested in ice-cold lysis buffer [100 mM tris–HCl (pH 8.3), 500 mM NaCl, 0.5% triton X-100, 1 mM DTT, and complete protease inhibitors (Roche)]. Cell lysates were incubated for 30 min at 4 °C and centrifuged at 20,000 × *g* for 30 min. The resultant supernatants were clarified by incubation with Dynabeads–Protein G (Invitrogen) for 1 h at 4 °C, and then anti-SUN2 pAb, anti-SUN1 pAb, or control IgG, and protein G beads were added to the lysates. The bound proteins were washed five times with lysis buffer, resolved by 10–20% SDS-PAGE, and silver stained or transferred to nitrocellulose.

### Mass spectrometry and data analysis

SUN2-containing protein complexes were purified, resolved by 10–20% SDS-PAGE, and stained with a mass spectrometry-compatible Silver Stain MS kit (Wako Pure Chemical Industries Ltd.). Corresponding bands were cut out for trypsinization. Tandem mass spectra of the trypsinized peptides were obtained using a nano LC (UltiMate 3000; Thermo Scientific Dionex, CA, USA) equipped with an L-column 2 C18 (150 mm × 0.075 mm i.d. analytical column, containing 3-µm particles; Chemicals Evaluation and Research Institute, Japan), coupled with a nano-ESI (electrospray ionization)-IT (ion trap)-MS (mass spectrometer) equipped with a nano-ESI ion source (HCTultra; Bruker Daltonik GmbH, Germany). The raw data files were analyzed using Mascot MS/MS Ion Search (Matrix Science, London, UK) and searched against the Swiss-Prot database of human proteins.

### Quantification of Golgi positioning in the Purkinje cells of Sun1-knockout mice

Sun1-knockout mice were produced as described previously^31^. The experimental procedures were reviewed and approved by the Institutional Animal Care and Use Committees of the National Health Research Institutes (NHRI-IACUC-098086-A) and all methods were performed in accordance with the relevant guidelines and regulations by the committee. We performed experiments in compliance with the ARRIVE guidelines (http://www.nc3rs.org.uk/page.asp?id=1357). Mice were housed in sterile cages maintained under 12-h light/dark cycles with controlled temperature and humidity. Mice were anesthetized in isoflurane, perfused with PBS followed by 4% paraformaldehyde in PBS. Mouse brains were dehydrolyzed and embedded in paraffin. Immunohistochemistry was performed using paraffin sections of 30-day-old mice. Tissue paraffin sections were deparaffinized using xylene for 40 min 3 times, and were rehydrated by subsequent incubation in 85% and 75% ethanol for 5 min each. Antigen retrieval was achieved by placing the slides in 100 °C citrate buffer, pH 6.0 for 1 h. Slides were rinsed with ddH_2_O and PBS successively, and were immersed with 1% Triton X-100 in PBS for 30 min. To prevent non-specific binding, slides were blocked with 1% BSA for 30 min at room temperature. Primary antibodies (anti- Golga2 1:100 Sigma-Aldrich HPA021799 and anti-Calbindin 1:500 Sigma-Aldrich C9848) diluted in PBS were applied, and were incubated at room temperature for 1.5 h^33^. After three washes with PBS, secondary antibodies (goat anti-rabbit Alexa Fluor 488 1:500 Invitrogen A-11008 and goat anti-mouse Alexa Fluor 568 1:500 Invitrogen A-11004) diluted in PBS were added, and were incubated at room temperature for 45 min. The nucleus was counterstained with Hoechst 33342 (1:10,000, Invitrogen H3570) and the slides were mounted with Prolong Gold antifade reagents (Invitrogen P10144). Images were recorded using a Leica TCS SP5 confocal microscope (Leica) equipped with HyD (hybrid detector). Golgi distribution was then quantified using GM130 staining as the average percentage of total cellular fluorescence in each of the four quadrants, as described^18^. In brief, the soma region was divided into quadrants and total cellular fluorescence in each quadrant was measured. The percentage of cellular fluorescence for the quadrant was calculated for each cell before averaging.

## Supplementary Information

Supplementary Information

## Abbreviations

NE: Nuclear envelope
INM: Inner nuclear membrane
ONM: Outer nuclear membrane
LINC complex: Linker of nucleoskeleton and cytoskeleton
MT: Microtubule
GM130: Golgi matrix protein 130
siRNA: Small interfering RNA
