# Supplementary material for: The SUN2-nesprin-2 LINC complex and KIF20A function in the Golgi dispersal
Source: Sci Rep. 2021 Mar 8;11:5358. doi: 10.1038/s41598-021-84750-4 (PMC7940470; doi:10.1038/s41598-021-84750-4)
Supplement: Supplementary file 1 — Supplementary Information [file 41598_2021_84750_MOESM1_ESM.pdf]

Supplementary information

Title: The SUN2-nesprin-2 LINC complex and KIF20A function in the Golgi dispersal

Miki Hieda, Taizo Matsumoto, Mari Isobe, Sadamu Kurono, Yuka, Kaneko, Satoshi Kametaka, Jing-Ya Wang, Ya-Hui Chi, Kenji Kameda, Hiroshi Kimura, Nariaki Matsuura, Shuji Matsuura

Supplemental Methods

Cell cycle analysis

Cells were transfected with indicated siRNA. After 48 h incubation, cells were collected and stained with PI using BD Cycletest Plus DNA kit (Becton, Dickinson and Company, CA, USA). The cells were then analyzed with Gallios flow cytometer, (Beckman Coulter, CA, USA).

Table S1. siRNA targeting sequence (all siRNAs were 3' end overhanged)

Name of siRNA	Target sequence	Obtained from and designed by
siSYNE1	GAAAUUGUCCCUAUUGAUU<br>GCAAAGCCCUGGAUGAUAG<br>GAAGAGACGUGGCGAUUGU<br>CCAAACGGCUGGUGUGAUU	Thermo Scientific (SMART pool)
siSYNE2	CCACAGAGCUCCAAAGUAG<br>GAGCAAGUGUCCCAAGAUU<br>GAACUAAUGCAACUGGAAA<br>AGGAAUUUCUGCAAACCGA	Thermo Scientific (SMART pool)
siSYNE3 a	UGAGGAAAGUUCUGGAGAA	Nippon Gene
siSYNE3 b	UCGCUACCAUCCAGGAGUA	Nippon Gene
siSYNE3 c	GCGGGACCUUCCUGGAAAA	Nippon Gene
siKIF20A	CUGAAGAGUUGCAUAAGUA<br>GCAAUGAGAUGGUAGAACA<br>GAAGAGGGCCAGAAGAAUA<br>GCAAGAACCUGCUAUCAGA	Thermo Scientific (SMART pool)

Table S2. Primer sequences used in this paper

Target	Primer 1	Primer 2	Amplicon (bp)
nesprin-1	GAGTCCCACATCAGGAAGGA	CTCGGAGGACTCTGAACAGG	188
nesprin-2	TCGGAGGGAACTAATGCAAC	CGAGTCTACCTCGTCGAAGC	250
nesprin-3	CCTGGGAGCAGCAGATTAAG	ACTCCTGGATGGTAGCGATG	273
KIF20A	AGTCAGTGGCCCATCAGCAATC	TCAACACAGTATGATACTGCTCA	474

Hieda et al., Supplementary Figure 1
low-power field of the Golgi complex in SUN1 depleted cells

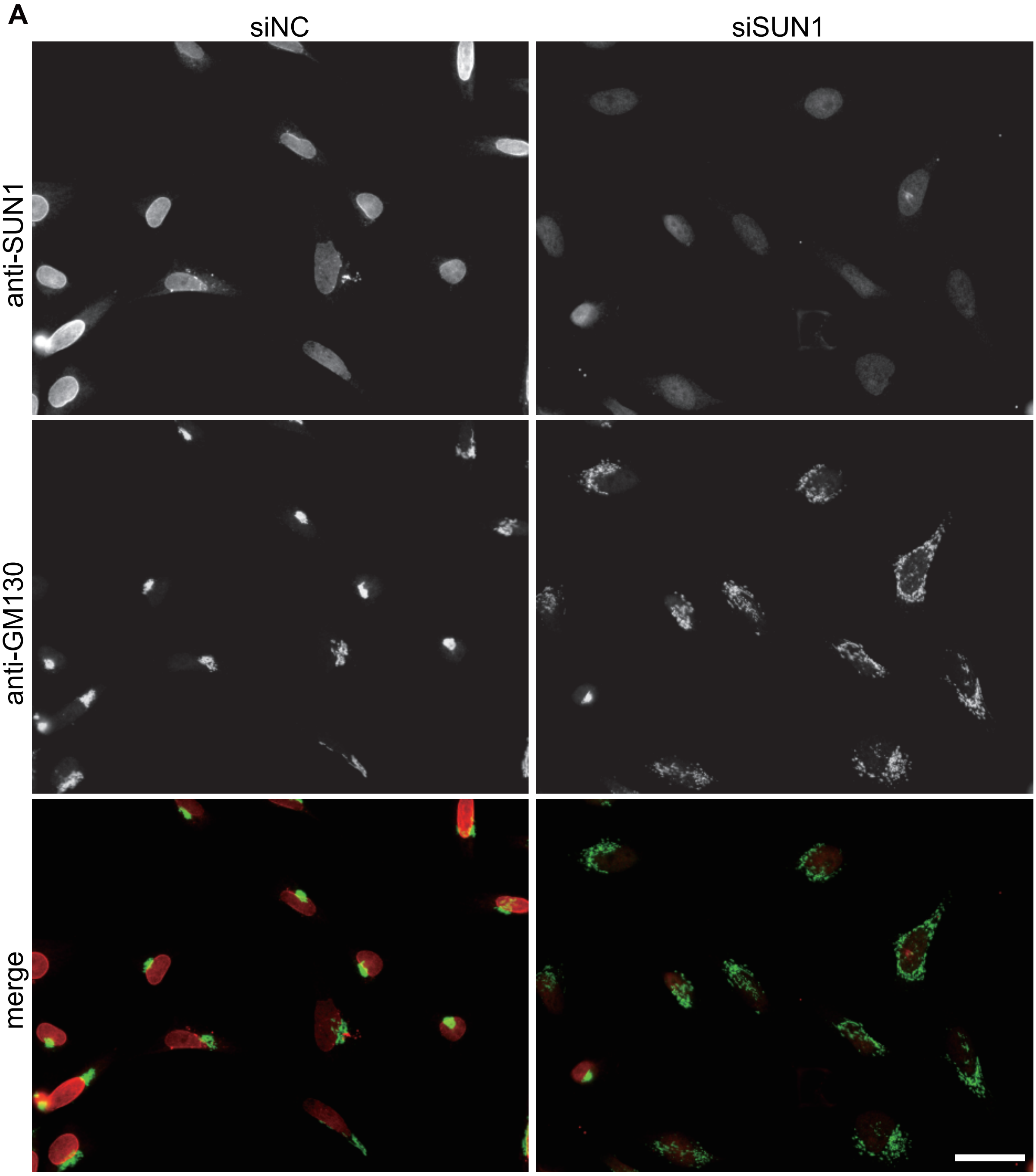

Hieda et al., Supplementary Figure 2
Effects of each siRNA against SUN1 and
Golgi complex morphology in SUN1 depleted MCF10A cells

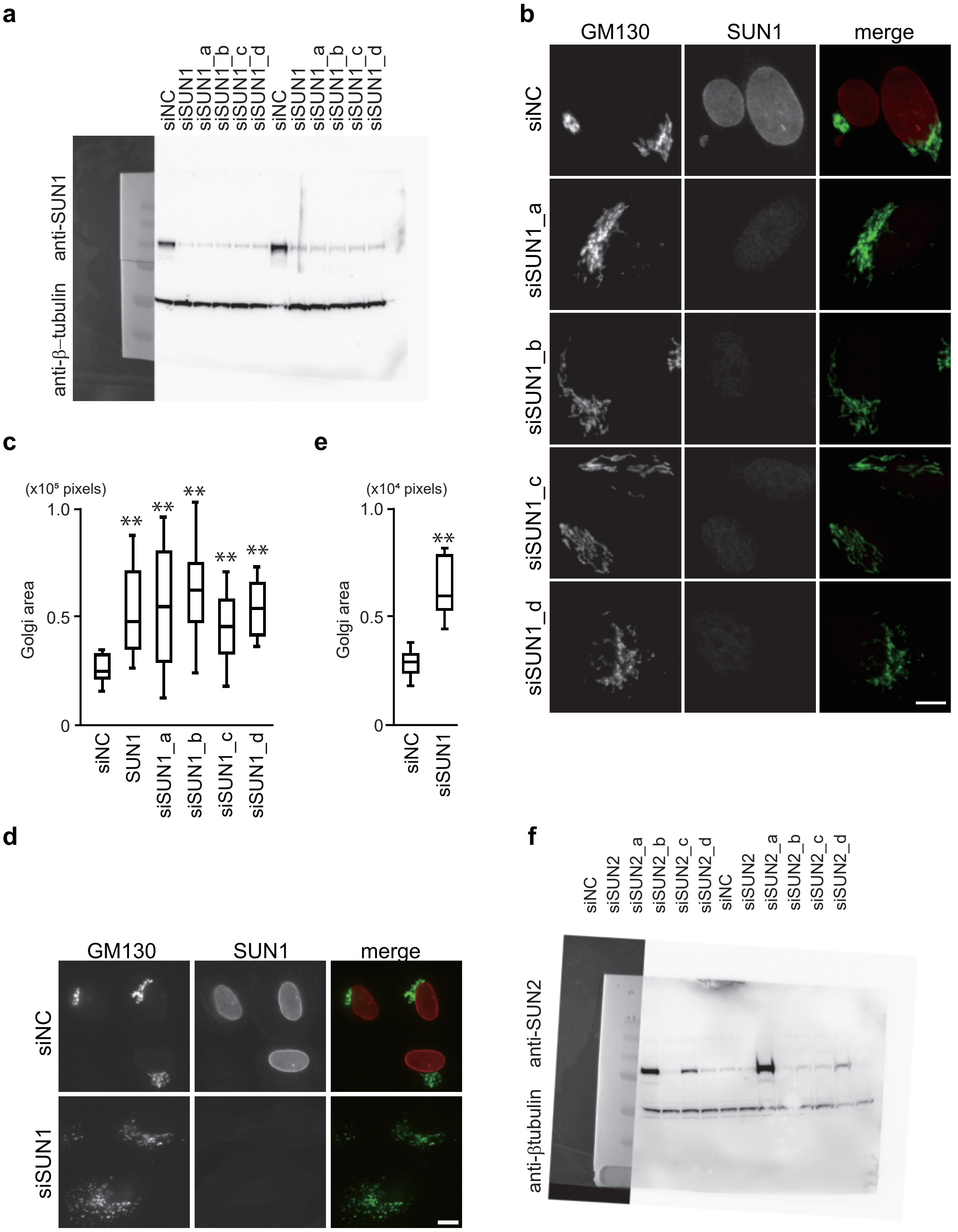

Hieda et al., Supplementary Figure 3

SUN1 and/or SUN2 does not affect the cell cycle

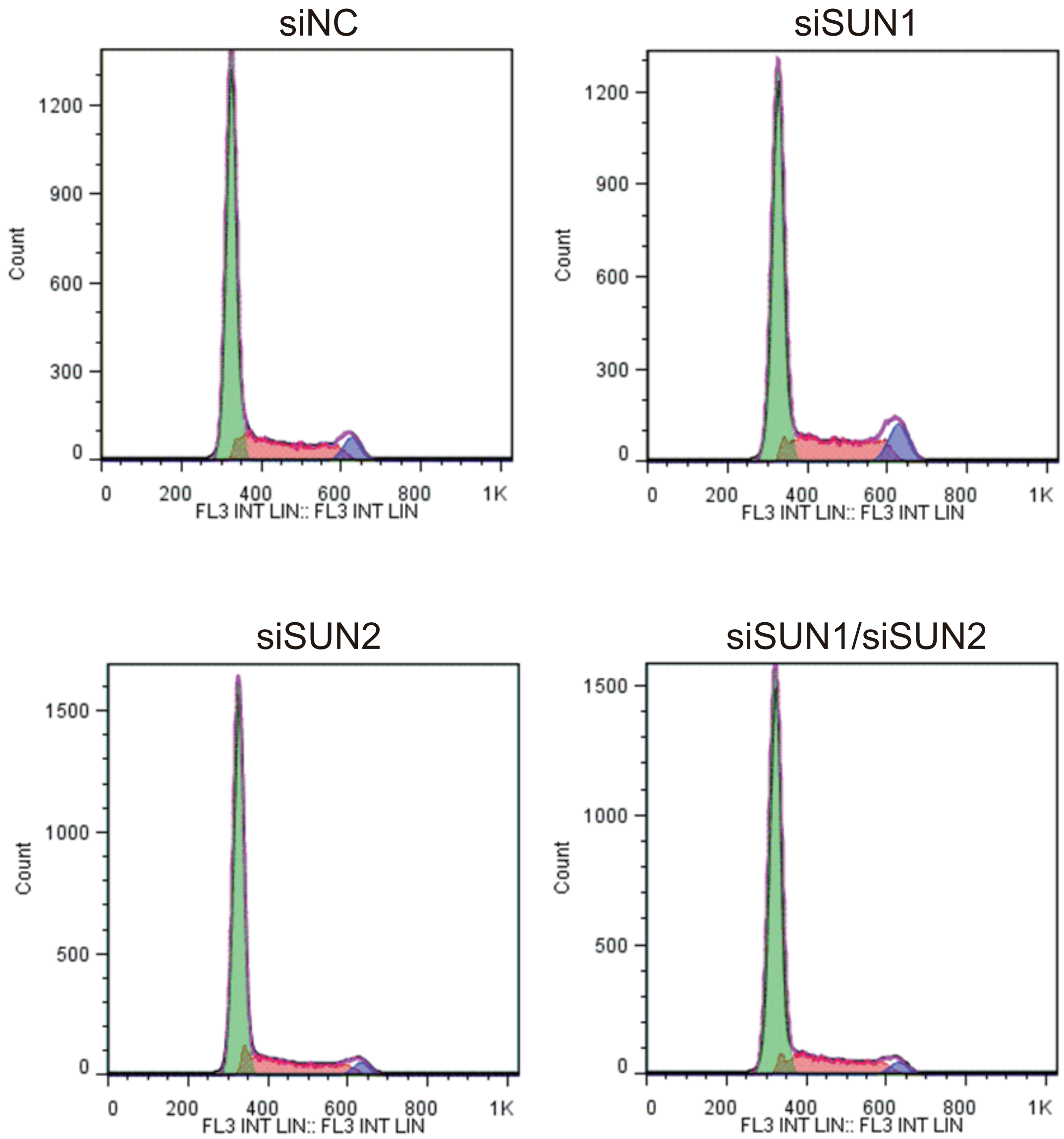

Hieda et al., Supplementary Figure 4

Knock down was confirmed by RT-PCR

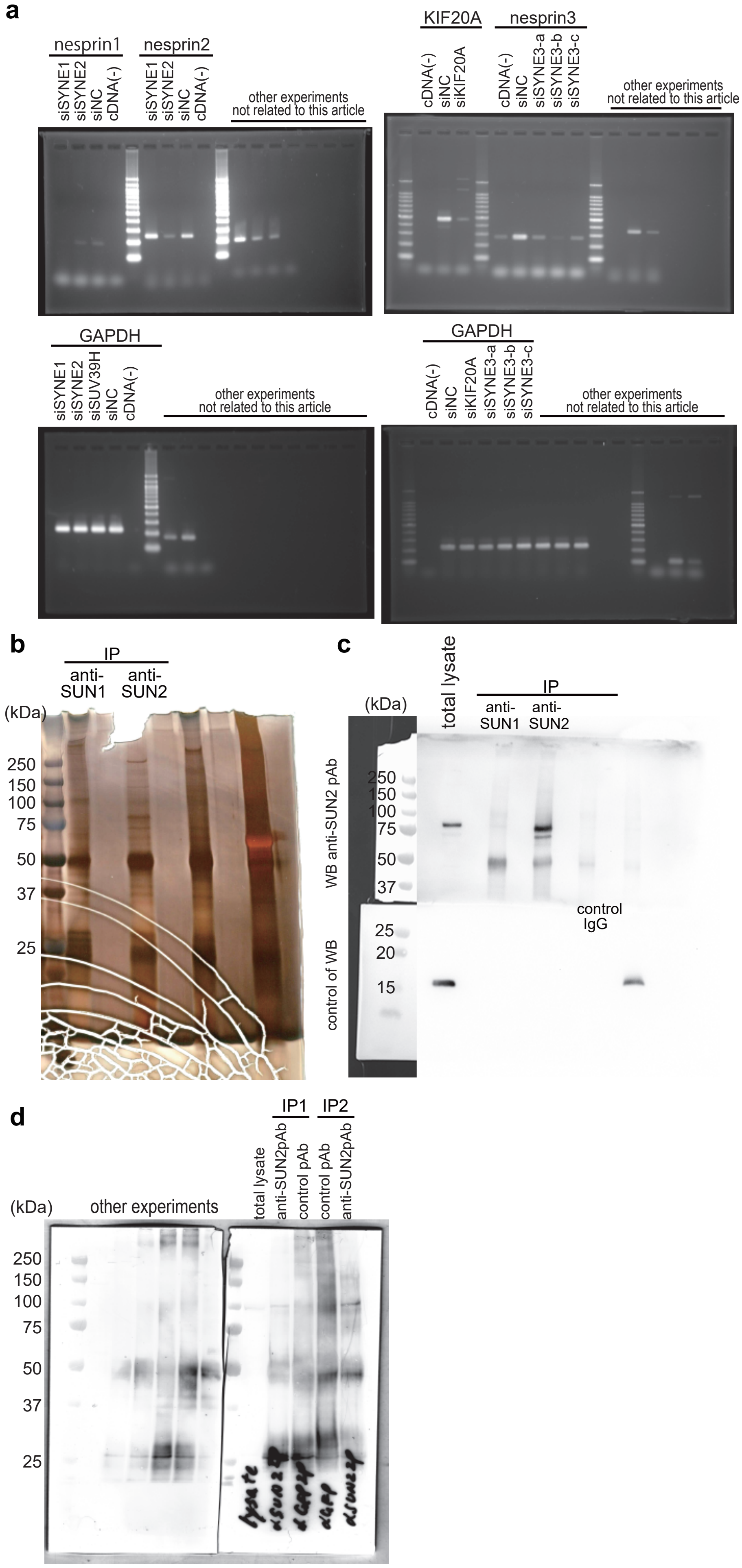

Supplementary Figure Legends

Figure S1. SUN1 associates with the Golgi complex morphology. Low-power field photos of the Golgi complex in SUN1-depleted cells. A pool of four kinds of siRNA against SUN1 (siSUN1) or negative control siRNAs (siNC) was transfected into HeLa cells. Each dish contains 2.5×10^5 cells. After 48 h incubation, cells were fixed and stained with anti-SUN1 pAb and anti-GM130 mAb. Bar, 20 μm.

Figure S2. Each single siRNA induced the Golgi dispersion and SUN1 depleted MCF10A cells show the dispersed Golgi complex. (a) Full size image of Figure 1.b. **(b)** HeLa cells were transfected with each single siRNA against SUN1 (SUN1_a, SUN1_b, siSUN1_c, and siSUN1_d) or siNC. After fixation, cells were stained with anti-GM130 mAb (green) and anti-SUN1 pAb (red). Bar, 10 μm. **(c)** Box-and-whiskers plots represent GM130 labeled Golgi complex area per cell**.** ** $P < 0.01$, compared with siNC transfected cells. **(d)** MCF10A cells were transfected with siSUN1 or siNC. After fixation, cells were stained with anti-GM130 mAb and anti-SUN1 pAb. **(e)** Box-and-whiskers plots represent GM130 labeled Golgi complex area per cell**.** ** $P < 0.01$, compared with siNC transfected cells. **(f)** Full size image of Figure 2b. The right half and the left half use the same lysate but different volumes were applied.

Figure S3. SUN1 and/or SUN2 depletion does not affect the cell cycle distribution. HeLa cells were transfected with indicated siRNA. After 48 h incubation, cells were collected and stained with PI. The cell cycle was then analyzed.

Figure S4. Knockdown efficiency of nesprins and KIF20A.
(a) HeLa cells were transfected with an siRNA mixture against nesprin-1, nesprin-2 (siSYNE1 or siSYNE2, respectively, Table S1), siRNA mixture against KIF20A, or each of three kinds of siRNA against nesprin-3 (siSYNE3-a, siSYNE3-b, and siSYNE3-c, Table S1). After 48 h incubation, RNA was collected and expression of nesprins was examined by RT-PCR. **(b) (c), and (d)** Full size images of Figure 4a, 4b, and 4c. **(e)** HeLa cells were transfected with an siRNA mixture against
